# Dietary Bioactive Compounds and Breast Cancer

**DOI:** 10.3390/ijms24119731

**Published:** 2023-06-04

**Authors:** Juan Antonio Giménez-Bastida, Antonio González-Sarrías

**Affiliations:** Laboratory of Food and Health, Research Group on Quality, Safety and Bioactivity of Plant Foods, Department Food Science and Technology, CEBAS-CSIC, Campus de Espinardo, 30100 Murcia, Spain

Cancer is among the most serious health problems and the second leading cause of death globally, affecting millions of people worldwide. Breast cancer (BC) is the most commonly diagnosed cancer type and the first cause of cancer-associated death in women and their incidence is dramatically growing together with increased lifespan, accounting for 1 in 8 cancer diagnoses worldwide [1]. The etiology of BC is attributed to a complex interaction between various modifiable factors including age, poor eating habits, smoking, and(or) lack of physical activity, among others, and non-modifiable factors or hereditary, which encompasses around 10–15% of all BC [2]. In this line, increasing epidemiological and observational studies suggest and support the idea that the adherence to plant-based diets are of great significance for the primary prevention of BC as a valuable source of natural biologically active compounds (beyond their nutritive value) including both bioactive nutritional components such as vitamins and minerals and non-nutritional ones such as polyphenols, among others; however, results obtained from individual supplements in humans are still inconsistent and insufficient to prevent BC or as an adjuvant to treat it. Additionally, despite the information available by numerous preclinical studies on the antiproliferative and(or) anticancer properties of different bioactive compounds associated with plant food consumption, the identification of their involvement and the underlying mechanism behind these activities remains contradictory [3]. In this context, a number of key points should be considered in the in vitro and in vivo research on dietary bioactives against BC: (i) avoid wrong approaches that do not consider the bioavailability, metabolism, and breast tissue distribution of dietary bioactives; (ii) increase the number of carefully designed clinical studies to overcome the main limitations (sample size, duration, inter-individual variability); (iii) identify the underlying molecular mechanisms and evaluate a broader range of cancer biomarkers. Thus, further studies need to overcome these critical limitations to assert the real bioactive metabolites and the potential mechanisms responsible for the efficacy of dietary bioactives on BC.

Therefore, according to what was previously stated, this Special Issue includes studies on the chemopreventive and/or chemosensitization effects on BC of dietary bioactive compounds and/or derived metabolites using physiologically relevant preclinical (cell and animal models) and clinical approaches, that could elucidate if they are (at least in part) responsible for the effects attributed to plant-based foods. This Special Issue provides three comprehensive review and three original articles focused on these realistic approaches to the detriment of enthusiasm of non-physiological studies that are still published nowadays as previously underlined by Ávila-Gálvez et al. (2018) [4]. However, there is still a huge gap between the promising preclinical results reported and their extrapolation into clinics.

The studies included in this Special Issue shed light in order to answer important questions such as “what” compounds exert the anti-carcinogenic effects and “how” these molecules act against BC. An example of this approach comes from a study by Codini et al. who reviewed the evidence reported in numerous (preclinical and clinical) studies published between 1950 and 2020 regarding vitamin C and BC [5]. Higher survival rate, improvement of quality of life, and reduction in the side-effects linked to chemotherapy are desirable effects observed in Vitamin C-consuming patients. Further in vivo studies highlight the inhibitory effect of vitamin C against metastasis, which could be the result of its capacity (described in vitro) to increase 5-hydroxymethylcytosine levels, target the immune system and the biosynthesis of important molecules such as prostaglandins (PGE_2_) and interleukins (IL-8 and IL-18), regulate gene expression (acting on transcription factors or DNA methylation) and(or) exert pro-oxidant effects on cancer cells [5]. Besides vitamins, phytoalexins (molecules formed in response to stress situations) are in the focus of food scientists due to their chemopreventive function. Within this group, glyceollins show an ability to inhibit the growth of BC cells (i.e., MCF-7 and T47Darom) thanks to their anti-estrogenic activity alone [6] or in combination with other pharmacological compounds, including lapatinib [7]. (Poly)phenols are another group of natural bioactive compounds widely investigated against BC. The first review published in this Special Issue provides an exhaustive and comprehensive analysis of the studies related to the effects (identification of biological targets and molecular mechanisms) of a wide range of (poly)phenols (i.e., resveratrol, quercetin, isoflavones, epigallocatechin gallate, lignans and curcumin) regarding the prevention/treatment of BC [8]. Additional investigations within this Issue focused on the cellular and molecular mechanisms of two groups of (poly)phenols such as methoxyflavones and stilbenes. Thus, chrysoeriol blocked the TNF-α-induced expression of CYP19 in MCF-7 BC cells [9]. Otherwise, pterostilbene, piceatannol and resveratrol exerted their effects through epigenetic regulation (DNA methylation, modulation of microRNA levels, and interaction with transcription factors and signaling pathways) [10] as well as synergic effects with radiotherapy (cytotoxicity, apoptosis induction via p53, caspases and Bax/Blc2 level modulation, among others) [11]. These studies overlook the idea that the phase-II conjugated metabolites (mainly glucuronides and sulfates) are the molecules detected in BC tissues [12]. Although the presence of the free forms of (poly)phenols is conceivable via deconjugation [13,14,15], physiologically relevant in vitro studies require the study of conjugated and free forms in parallel [16,17]. This is critical to reproduce the promising results from preclinical studies in clinical trials carried out in BC patients [4]. 

In conclusion, this Special Issue includes reviews that summarize and update the relevant knowledge evolved from the field of dietary bioactive compounds and BC treatment/prevention. It also includes research studies describing new cellular and molecular mechanisms by which natural molecules exert their chemopreventive effects. Hence, the information described in this Isue paves the way of desiging new relevant preclinical studies, which might contribute to carrying out future well-designed (and highly robust) clinical trials and establishing preventive nutritional strategies. 
